# Modeling the Impact of Vaping: What We Need to Know and Which Methods to Use

**DOI:** 10.1093/ntr/ntae204

**Published:** 2024-09-03

**Authors:** Lion Shahab

**Affiliations:** Department of Behavioural Science and Health, University College London, London, UK; SPECTRUM Consortium, Edinburgh, UK

Recent articles in *Nicotine & Tobacco Research* highlight the uncertainties that remain about the impact of vaping on population health.^1^ E-cigarettes generally compete in a marketplace that allows the sale of cigarettes. Cigarettes remain the primary preventable cause of avoidable deaths and morbidity, exerting an enormous burden on population health worldwide.^2^ By contrast, while not completely safe, e-cigarettes are likely much less harmful as they do not contain tobacco and their use does not involve combustion, resulting in much lower levels of exposure to toxic or carcinogenic constituents.^3^ Models to estimate the effect of vaping on population health have to assess the impact of e-cigarette use on both smokers and nonsmokers. Generally, such models^1^ specify at least three key parameters (with subset scenarios) to be evaluated.

First, we need to know the *actual* health effects, for example, cancer incidence, as opposed to *likely* health effects (assessed by exposure or potential harm biomarkers) of e-cigarette use. Of note, while nicotine (as the primary addictive component in cigarettes and e-cigarettes) is a special case, having effects on both physical and mental health that may not be uniform across all ages, it is beyond the remit to discuss here. Actual health effects then must be assessed across three scenarios: (1) vaping versus using nothing (absolute effect), (2) vaping versus smoking cigarettes (relative effect), and (3) dual-use (vaping and smoking) versus smoking cigarettes (relative combined effect). As e-cigarettes—unlike cigarettes—do not produce side-stream emissions, and aerosol contains much lower levels of harmful excipients than cigarette smoke,^4^ the impact of secondhand exposure from e-cigarette use on population health is likely very small, possibly negligible, and not considered here. Second, we need to know the behavioral effect that e-cigarettes have on smokers. There are again three scenarios, with individuals transitioning between these: e-cigarette availability may result in (1) co-use of cigarettes and e-cigarettes (dual use), (2) permanent switching from cigarettes to e-cigarettes (harm reduction), or (3) stopping cigarettes and then e-cigarettes (cessation). Third, we need to know the behavioral effect that e-cigarettes have on current nonsmokers. Here we must distinguish never from past users, as past use likely impacts transition probabilities. There are three main scenarios: E-cigarette availability may lead to (1) starting e-cigarette use (uptake), (2) sequential use of e-cigarettes and then cigarettes, or (3) concurrent/dual use of e-cigarettes with cigarettes (both [2] and [3] constitute what is termed the “gateway in” hypothesis for never smokers, and “relapse” for past users). Figure 1 summarizes these pathways. Notably, while the direct transition between non-users and smokers does not involve e-cigarettes, it may be impacted indirectly, if e-cigarette availability either de- or re-normalizes smoking,^6^ thereby de- or increasing the likelihood of starting or continuing to smoke.

**Figure 1.**
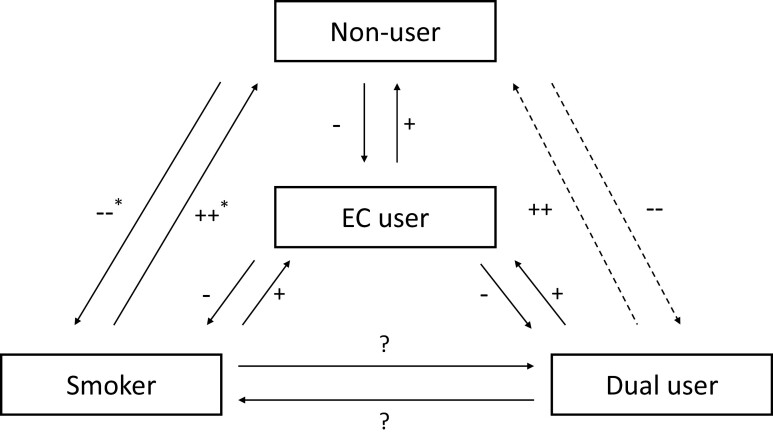
Individual-level transition pathways. EC = E-cigarette; *Indirect effect of e-cigarettes via de-or re-normalization of smoking; Non-users—this includes both past and never users of products; On the assumption that e-cigarettes are less harmful than cigarettes but not completely harmless, likely health effects and relative magnitude associated with transitions are presented in gradation (−/− − negative health impact; +/++ positive health impact;? unclear due to lack of clear evidence for a whole class of “dual users”); Transition probabilities are not directly presented as these requires empirical validation and line thickness is not meant to represent strength of probabilities. However, arrows between nonuse and dual-use are dotted as this state transition is likely less common than others, given that most people do not start or stop using several nicotine products simultaneously (eg,^5^).

Data collection to estimate parameters, and the population impact of vaping, is complicated by some key challenges: (1) most (tobacco-related) diseases take a long to develop (eg, around 20-30 years for lung cancer)^7^; (2) people frequently change behavior (eg, switching between e-cigarettes and cigarettes or different e-cigarette types and other (combustible/noncombustible) tobacco products) producing complex exposure profiles (especially for dual users)^8^; (3) e-cigarette use is not as easily quantified as cigarette consumption, complicating assessment of exposure; (4) e-cigarettes encompass different categories (eg, disposables/reusables, open/closed) and e-liquids (eg, protonated/unprotonated nicotine), making the assessment of the impact of individual product characteristics difficult; (5) the e-cigarette market, and regulation of it, is ever-changing, requiring real-time monitoring of effects.

While causal pathways are commonly estimated with randomized controlled trials (RCTs), given the above challenges, RCTs are not particularly useful here. First, for several comparisons (those with non-users), it would be unethical to randomize people to using an e-cigarette or not (eg, to estimate health or gateway effects). Second, given our interest in long-term health outcomes and complex behavioral transitions, RCTs would be impractical because of the need for large sample sizes and long follow-ups. Third, RCTs often have limited external validity,^9^ not reflecting the real-world effects needed here. Finally, RCTs are relatively inflexible and would not be able to capture the dynamic impact of a changing e-cigarette marketplace. Observational studies, even if they do not proffer strong causal inferences, are therefore better for estimating most parameters required to assess the population impact of vaping. There are two broad types of observational data sources (individual-level and population/aggregate-level) with different study types we can draw on (Table S1).

For health effects, longitudinal cohort studies, using individual-level data assessing vaping and smoking over time, can capture some of the complexities in exposure and estimate associations with actual health outcomes through linkage with health records over the long term, as the British Doctors Study did.^10^ This approach has the advantage that associations with many different health outcomes can be evaluated simultaneously, including with novel outcomes that have not been associated with tobacco use (“unknown unknowns”). A disadvantage is the length of time it takes to collect data and get answers. Another common individual-level approach is the use of case–control studies where cases are selected based on a given outcome (eg, hospitalization with a tobacco-related disease) and compared with controls (eg, hospitalization with a tobacco-unrelated disease) on exposure to putative risk factors (eg, vaping), as happened in early lung cancer studies.^11^ Case–control studies are quick, but assess only one disease at a time, and “unknown unknowns” are unlikely to be discovered.

For behavioral transitions, in smokers at least, RCTs can help provide causal estimates for short- to medium-term effects.^12^ For long-term effects, complex transitions and those involving non-users, individual-level analyses, discussed above, can be undertaken, for example, applying structural equation modeling to cohort studies to estimate bi-directional associations between various behavioral states. However, a complicating factor is the possibility that longitudinal associations between behaviors (vaping and smoking) may, in fact, reflect common liability (eg, predisposition to engage in risky behavior),^13^ rather than causal effects. Here, instrumental variable analysis with an instrument causally related to e-cigarette use but without any plausible causal connection with smoking (or vice versa) can be employed to provide relatively unbiased estimates, for example, using genetic variants as proxies for exposure in Mendelian Randomization studies.^14^ The main advantage is that stronger causal inferences can be drawn, but a problem is that identifying suitable instruments may be difficult. A final issue to consider is that not all behavioral effects of e-cigarettes can be directly observed and evaluated at the individual level. For instance, while it may be true for some that e-cigarettes cause relapse or act as a gateway into smoking, for others the opposite may be the case, with e-cigarettes diverting them away from relapsing or starting to smoke,^15^ or, indeed, delaying smoking initiation. Estimating this effect would require a counterfactual scenario where e-cigarettes are not available. As individuals may move in either direction, what we really need for estimating the population impact is a net effect. Population-level approaches are best suited here and have an additional advantage over individual-level analyses because they are not biased by individual-level confounding.

Quasi-experimental designs, including natural experiments, offer a useful population-level method to examine the effects of vaping by exploiting naturally occurring differences (eg, in policy environments restricting e-cigarettes in one but not another locality), while other environmental influences remain unchanged. An advantage is that the net effect on multiple outcomes can be assessed quickly at the same time (eg, for smoking rates and health outcomes) using historical data, but a drawback is that policy changes rarely just impact one driver (ie, vaping) that influences outcomes of interest. This can result in nonequivalent confounding structures between populations post-policy change, which would need to be considered in the analysis. Another option is the use of complex systems models,^16^ where postulated effects (eg, of e-cigarette use on transition probabilities or disease incidence), at the micro- or macro-level, or both, are used to generate hypothetical data, which can be compared with observed data to evaluate whether parameter estimates are likely. An advantage is that counterfactual scenarios can be tested, and granular associations estimated; a drawback is that the model’s veracity will depend on correct inputs and calibration (which becomes more difficult with increasing model complexity). Lastly, using aggregate data, multiple timeseries analyses can determine the association of an exposure timeseries (eg, vaping prevalence) with an outcome timeseries (eg, disease incidence), controlling for established population-level confounders and accounting for seasonal variations, autocorrelation, and removing underlying trends. These have been used to study the real-world effects of e-cigarette use on smoking cessation rates^17^ or of tobacco bans on heart disease.^18^ The main advantage is that—depending on the timeseries length and quality of data—longer-term effects (eg, for more acute cardiovascular health effects) can be assessed relatively cheaply now, as existing nationally representative data sources have captured e-cigarette use for over a decade already. The main drawback is that exposure and outcome measures are not assessed at a granular, detailed level and thus smaller, potentially important, effects or more complex associations may be missed, and that long-term effects will not capture, and thus generalize to, current use patterns and newer products.

Many other designs and data sources (for a comprehensive list see^19^) exist that can help estimate the required parameters pertaining to the health consequences of vaping, and its impact on behavior change among smokers and non-users. However, as each approach has unique advantages, drawbacks, and biases, it is likely that formal triangulation of data sources and methods^20^ will provide the most complete answer to evaluate the population impact of vaping. Now is the time to make use of these varied methodologies to improve our knowledge of the impact of vaping on population health.

## Supplementary material

Supplementary material is available at *Nicotine and Tobacco Research* online.

ntae204_suppl_Supplementary_Materials
