# Supplementary material for: Modeling the Impact of Vaping: What We Need to Know and Which Methods to Use
Source: Nicotine Tob Res. 2024 Sep 3;27(3):561–3. doi: 10.1093/ntr/ntae204 (PMC11847779; doi:10.1093/ntr/ntae204)
Supplement: ntae204_suppl_Supplementary_Materials [file ntae204_suppl_supplementary_materials.docx]

**Supplementary Table 1: Examples of different designs and data sources to assess population impact of e-cigarettes**

|  | ***Design*** | ***Example*** | ***Key biases*** | ***Advantages*** | ***Drawbacks*** |
| --- | --- | --- | --- | --- | --- |
| ***Individual-level data*** | | | | | |
| Cohort studies with linkage to health care records | Longitudinal | Multivariable log-binomial regression: longitudinal survey to assess association between e-cigarette use across lifetime (e.g., measured combining amount per year*years used or simply as yes/no) among never smokers and incidence of specific disease over follow-ups using multilevel models, generalized estimation equations and others. | Residual and unmeasured confounding of individual-level influences; systematic attrition | - Looks at many exposures and outcomes at once - May identify unknown exposure-outcome associations - Excludes reverse causation - Adaptable to changes over time in environment | - Takes long time - Costly - Requires large samples - Low power for rare outcomes |
| Case-control studies | Cross-sectional | Multivariable logistic regression: hospitalised cases with outcome of interest (e.g., incident respiratory disease) are compared with similar hospitalised controls without outcome of interest (e.g., with arm fractures) in terms of vaping and smoking history (no smoking, no vaping, past smoking, past vaping, current smoking past vaping and so on) to estimate odds ratio of exposure combination with outcome of interest | Recall bias, sampling bias from unmeasured confounding; not useful for rare risk factors. | - Fast - Relatively cheap - Small sample - Good for rare outcomes | - Looks at only one outcome - Temporal sequence effects/complex exposure difficult to disentangle - Not adaptable - provides snapshot only |
| Instrumental variable analysis | Cross-sectional; longitudinal | Mendelian randomisation (MR): Use of a specific genetic variant linked with e-cigarette use (but not smoking behaviour) to look at association with smoking in those with and without the genetic variant. If association is similar, e-cigarettes are unlikely to cause smoking but if the association is stronger in those with variant, it likely reflects some causal effect of e-cigarette use on taking up smoking. | Weak associations between the instrument and the exposure can introduce bias; exclusion restriction criterion violation (due to horizontal pleiotropy) and population stratification are main sources of bias in MR | - Stronger causal inferences can be drawn (avoids reverse causation and residual confounding) - Use of retrospective data means efficient, timely analysis possible - Multiple outcomes (behavioural and health-related) can be assessed | - Difficult to identify good instruments - Limited availability of good data (for genetic studies) - Can require relatively large sample sizes |
| ***Population-level data*** | | | | | |
| Natural experiment | Repeat cross-sectional | Several statistical methodologies can be applied, but the main drive is that differences in a desired outcome are compared for populations that are exposed to variations introduced in the environment, but which have otherwise a similar confounding structure. For instance, changes in smoking rates over time are compared in countries where a ban on e-cigarettes is introduced compared with changes in countries with liberal access to e-cigarettes. | Unmeasured confounding resulting in non-equivalent populations; no proper segmentation of exposure (e.g., policy violations); potential selection bias; measurement differences across populations. | - Fast - Cheap - Avoids individual confounding - Can look at several outcomes at once | - Risk of population-level confounding due to potentially complex impact of ‘quasi-random’ variation beyond target exposure - Smaller, important effects missed - Not suitable for rare exposures/outcomes |
| Complex systems models* | N/A | A microsimulation model calibrated on data from a population level survey that assigns different individual transition probabilities from not smoking to smoking based on variety of individual level characteristics, including e-cigarette use, to forecast changes in smoking prevalence and compare with observed smoking rates. The postulated impact of e-cigarette use on individual state transitions can be varied to provide best fit to data. | Model misspecification (relevant confounders not included); results being only as good as the parameter estimates used and inputs included. | - Can handle counterfactual scenarios - Can assess very granular, complex phenomena and produce unpredicted, emergent outcomes (‘unknown unknowns’) - Fast as does not require data collection - Can be used in long-term forecasting | - Can be computationally complex - Can be difficult to interpret (‘black box’) - Model calibration and validation can be challenging c |
| Multiple time series analysis | Repeat cross-sectional | Auto-Regressive Integrated Moving Average with eXogenous inputs (ARIMAX) model: Assess whether changes in the prevalence of e-cigarette use among never smokers is associated with incidence rates of myocardial infarction in never smokers, adjusting for known population-level confounders (e.g., influenza incidence, air pollution). | Model misspecification, unmeasured confounding at population level. | - Fast - Cheap - Avoids individual confounding - Can look at longer time horizon now, without risk of recall bias - Can account for multiple population-level confounders | - Cannot disentangle complex exposures - Low power so may miss smaller, important effects - Not suitable for rare exposures/outcomes - Looks at single outcomes |

*Can be at both individual and population level
